# Pharmacies in informal settlements: a retrospective, cross-sectional household and health facility survey in four countries

**DOI:** 10.1186/s12913-021-06937-9

**Published:** 2021-09-09

**Authors:** Improving Health in Slums Collaborative, Pauline Bakibinga, Caroline Kabaria, Ziraba Kasiira, Peter Kibe, Catherine Kyobutungi, Nelson Mbaya, Blessing Mberu, Shukri Mohammed, Anne Njeri, Iqbal Azam, Romaina Iqbal, Ahsana Nazish, Narijis Rizvi, Syed A. K. Shifat Ahmed, Nazratun Choudhury, Ornob Alam, Afreen Zaman Khan, Omar Rahman, Rita Yusuf, Doyin Odubanjo, Motunrayo Ayobola, Olufunke Fayehun, Akinyinka Omigbodun, Mary Osuh, Eme Owoaje, Olalekan Taiwo, Richard J. Lilford, Jo Sartori, Samuel I. Watson, Peter J. Diggle, Navneet Aujla, Yen-Fu Chen, Paramjit Gill, Frances Griffiths, Bronwyn Harris, Jason Madan, Helen Muir, Oyinlola Oyebode, Vangelis Pitidis, João Porto de Albuquerque, Simon Smith, Celia Taylor, Philip Ulbrich, Olalekan A. Uthman, Ria Wilson, Godwin Yeboah, Sam Watson

**Affiliations:** Institute of Applied Health Research, University of Birmingham, Birmingham, UK

**Keywords:** Healthcare access, Pharmacies, LMICs, Slums, Welfare

## Abstract

**Background:**

Slums or informal settlements characterize most large cities in LMIC. Previous evidence suggests pharmacies may be the most frequently used source of primary care in LMICs but that pharmacy services are of variable quality. However, evidence on pharmacy use and availability is very limited for slum populations.

**Methods:**

We conducted household, individual, and healthcare provider surveys and qualitative observations on pharmacies and pharmacy use in seven slum sites in four countries (Nigeria, Kenya, Pakistan, and Bangladesh). All pharmacies and up to 1200 households in each site were sampled. Adults and children were surveyed about their use of healthcare services and pharmacies were observed and their services, equipment, and stock documented.

**Results:**

We completed 7692 household and 7451 individual adults, 2633 individual child surveys, and 157 surveys of pharmacies located within the seven sites. Visit rates to pharmacies and drug sellers varied from 0.1 (Nigeria) to 3.0 (Bangladesh) visits per person-year, almost all of which were for new conditions. We found highly variable conditions in what constituted a “pharmacy” across the sites and most pharmacies did not employ a qualified pharmacist. Analgesics and antibiotics were widely available but other categories of medications, particularly those for chronic illness were often not available anywhere. The majority of pharmacies lacked basic equipment such as a thermometer and weighing scales.

**Conclusions:**

Pharmacies are locally and widely available to residents of slums. However, the conditions of the facilities and availability of medicines were poor and prices relatively high. Pharmacies may represent a large untapped resource to improving access to primary care for the urban poor.

**Supplementary Information:**

The online version contains supplementary material available at 10.1186/s12913-021-06937-9.

## Introduction

Informal settlements and slums are home to a large and growing proportion of the world’s population: approximately one billion people are estimated to live in these neighborhoods, [1] almost exclusively in low and middle income countries (LMICs) [2, 3]. While various definitions of a slum exist, all invariably are based on exposure to hazards to human health and poor living standards either in the household or local environment [4]. Slum residents are also likely to face significant barriers when accessing healthcare [2, 3]. There is thus a priority to improve access to high quality health services for people living in slums, particularly in the context of achieving Universal Health Care (UHC).

Strengthening primary care has been described as the most “inclusive, effective, and efficient approach” to improving population health and wellbeing as part of achieving UHC globally [5]. “Primary care” is broadly defined as the first point of contact a patient makes with a health system and can include outpatient consultations with doctors and nurses, specialist providers, traditional and faith-based practitioners, and pharmacies – the subject of this article. Pharmacies provide medicines prescribed by doctors and nurses but they are also frequently the first and only place of contact when a health need arises and as such are often recognized as performing a wider public health role [6–8]. A well-cited example is the use of pharmacies to purchase anti-tussives by individuals presenting with chronic cough; pharmacies can play a key role in referring patients with suspected TB [8]. In Bangladesh, previous evidence has shown that slum dwellers make extensive, and often exclusive, use of pharmacies relative to other providers for a range of conditions [9].

A systematic review of pharmacy and drug store performance in LMICs in Asia found evidence of widespread inadequacies in services including inappropriate and illegal prescribing and dispensing of drugs, lack of referral, and a lack of information provision to customers [10] Similar results were also found in Sub-Saharan Africa [11]. These characteristics are often further combined with high prices, which are unaffordable for many [12–14].

Given the potentially frequent use of pharmacies and risk of low-quality care, they are therefore an obvious target to improve healthcare for the urban poor. Educational and quality improvement interventions have been shown to improve pharmacy practice [15]. However, the evidence is fairly limited on the availability and use of pharmacies and drug stores, the variability in what constitutes a pharmacy in different urban settings, and the availability of equipment, staff, and medication in those facilities. The role that pharmacies can play in improving access to high-quality healthcare, and how they should be engaged with from a policy perspective [16–20], strongly depends on their capacity to provide services and their available equipment and expertise. We therefore examined the availability and use of pharmacies among the urban poor, archetypically represented by those living in informal settlements. In each of seven slum sites across Nigeria, Kenya, Pakistan, and Bangladesh, we observed the distribution of pharmacies, the type of business and medicines stocked by each pharmacy, and we estimated use rates among the local population. We hypothesized that we would find relatively frequent use of pharmacies for both pre-existing and new conditions, and that the available services would be poor quality.

## Methods

### Setting

The aim of this study was to examine pharmacies available to urban slum dwellers from both the supply and demand perspectives. The data analyzed for this article were collected as part of a larger programme of work examining health and healthcare in informal settlements, the methods of which are described in detail elsewhere [21]. The surveys investigated use of a wide range of providers including outpatient doctor and nurse care, specialist care, inpatient care, and traditional and faith-based practice. Analyses of other types of care are reported elsewhere. We describe the specifics of the methods here relevant to the reported results. Legally there are distinctions between “drug vendors”, “patent medicine sellers”, pharmacies, and other types of facilities in each of our countries, although all provide pharmacy services. In the absence of an operationalizable set of definitions, we categorized any facility meeting the definition “A facility used for the compounding, dispensing of medicinal preparations, and/or other associated professional and administrative services” as a pharmacy, and investigated the heterogeneity of products and services available within this category.

### Study population

The study was conducted in seven informal settlements in four countries. The sites, which we label anonymously, were in Nigeria (NG1, NG2, NG3), Kenya (KE1, KE2), Pakistan (PK1), and Bangladesh (BD1). The sites were chosen on the bases that they: (1) fulfilled the UN definition of a ‘slum’ and (2) were named, geographically distinguishable, neighborhoods within city boundaries. Each site occupied a distinct geographical area that defined the boundaries in which we surveyed all pharmacy facilities and sampled households and individuals, as described below.

### Study design and procedures

Three datasets were collected comprising quantitative supply and demand side observations, and qualitative observations of the pharmacy facilities.

#### Health care facilities survey

All structures in each site were first mapped using Very High Resolution satellite imagery, and then “ground-truthed” through participatory mapping and corrected as required. All households in each structure were identified, which formed the sampling frame for the household survey described below. Non-residential structures were identified as such and we determined if they housed health care facilities. Field workers later returned to complete a comprehensive facilities survey in each location. We classified and defined facilities according to the schema in Table S2 (Supplementary Information) adapted from the American Medical Association’s taxonomy, local knowledge, and other sources [22]. A health facility could meet multiple definitions depending on its services and data were collected relevant to its classification(s).

Health care facility surveys aimed to capture the available services, human resources, products, and tests and diagnostic facilities available at each facility. Questions were facility type dependent. For pharmacies, the availability of medicines on the WHO list of essential medicines in the specific formulations was assessed [23], as well as basic medical equipment, such as weighing scales and latex gloves (detailed below). The availability of qualified medical personnel was assessed at all facilities; different grades of qualification were country specific. For pharmacies, we collected data specifically on qualified pharmacists and “pharmacy technicians” and related support staff. We also identified opening hours and costs where possible. Table 1 summarises the criteria used to characterize the facilities. We aimed for our list of criteria to be comprehensive and cover all the possibilities that a facility might reasonably have.

**Table 1:** Evaluation of available pharmacy products and services

We summarized the available equipment and services at each pharmacy according to the following criteria. Within each category the maximum score was one. **Essential medication** Within each category we totaled the number of the medications available (1 point if available, 0.5 points if available in a different dose or formulation) and divided by the number of drugs in the category. ***Antibiotics (4)*** Ciprofloxacin capsules (500 mg), co-trimoxazole suspension (8 + 40 mg/ml), amoxicillin capsules or tablets (500 mg), ceftriaxone injection (1 g/vial). ***Analgesics (2)*** Diclofenac capsules (150 mg), paracetamol suspension (125 mg/ml) ***Non-antibiotic medications for communicable diseases (6)*** Fluconazole capsules or tablets (150 mg), albendazole (400 mg) or mebendazole (100 mg or 500 mg) chewable tabs, oral rehydration salts (ORS) sachets, artemisinin combination therapy, isoniazid tablets (100 to 300 mg), fansidar (SP, sulfadoxine + pyrimethamine) tablets (500 mg + 25 mg) ***Medications for chronic illnesses (6)*** Salbutamol inhaler (0.1 mg/dose), glibencamide capsules or tablets (50 mg), atenolol capsules or tablets (50 mg), captopril (25 mg) or enalapril (2.5 mg) capsules or tablets, simvastatin capsules or tablets (20 mg), Efavirenz + lamivudine + tenofovir tablet (TDF + 3TC + EFV) (or relevant national standard for HIV) ***Medications for sexual health, family planning, and pregnancy (4)*** Oxytocin injection (10 IU in 1 ml ampule), combined oral contraceptive pills, medroxyprogesterone (25 mg) injectable contraceptive, male condoms. ***Other medications (3)*** Amitriptyline capsules or tablets (25 mg), omeprazole capsules or tablets (20 mg), vitamin A capsules (200,000 IU). **Medical equipment** Within each category we totaled the number of the items available and divided by the number of items in the category. ***Drug preparation and storage (4)*** Autoclave for sterilization, infusion kits for intravenous solution, refrigerator, latex gloves. ***Basic diagnostic equipment (7)*** Microscope, slides, sphygmomanometer, stethoscope, adult weighing scale, weighing equipment for under fives, thermometer. ***Advanced equipment (3)*** X-ray machine, oxygen system/cylinders, cytoflowmeter. **Pharmacy staff** For each category the pharmacy scored 1 for one or more full time staff member and 0.5 for one or more part time staff member. ***Qualified pharmacist (with or without degree certificate)*** ***Pharmacy technician (staff with lower-level qualifications)*** **Opening hours** The proportion of the week the pharmacy is open (out of 14, with each day divided into AM/PM). **Overall summary (**Fig. 2**)** An overall summary score summing all items in the medication, equipment, and staff categories was also calculated.	

#### Qualitative observations of health care facilities

Qualitative observation was undertaken at each facility surveyed, by two or three fieldworkers trained in the ethics and techniques of the method. An observation guide directed them to observe place, people, and activities and to note their overall impressions. Fieldnotes were taken on the health facility environment, structure, services advertised, apparent stock levels and activities at the time of observation. Observations were recorded in situ as handwritten notes that were expanded, transcribed, translated and digitized daily. These were summarized thematically and compared across sites.

#### Household and individual surveys

From the sampling frame of households described above, we used an inhibitory sampling design with close pairs to generate a spatially-regular and well-dispersed sample of 1200 households for each study site [24], assuming a response rate of 80%. Within each household, all residents were identified and an adult over 18 and a child under 12 were selected at random for the individual surveys. Adult women were oversampled compared to men at a ratio of 2:1 as we expected greater healthcare use among women. Up to three attempts were made to complete each survey should the participant not be available at the initial attempt.

Three instruments were used in the household-based surveys: (i) a household level survey containing a household roster, demographic, and socioeconomic questions including those used to construct the Demographic and Health Survey (DHS) wealth index [25]; (ii) an individual adult survey enquiring about health care need, access, and use, and health and well-being; and (iii) a child (under 12 years of age) survey asking a caregiver about health care needs, access, and use. Questions were adapted from similar studies to facilitate comparability [26]. The adult and child surveys both asked the respondents to provide details of the last time they used healthcare (if they had done so in the previous 12 months), including facility type (pharmacy, clinic, hospital, etc.), who they saw (doctor, nurse, pharmacist, etc.), the reason for the visit, the cost and time taken for the visit, and questions regarding their satisfaction with the care provided.

The data were collected on Android tablet devices using Open Data Kit software [27]. Survey instruments were translated using an iterative process involving forward and independent backward translations until a version was agreed and were then delivered in the local language.

#### Quality control

The data were quality controlled in the field, by field supervisors, using spot checks and direct observation. Data submitted to the server was inspected and cleaned and any errors or mismatching identifiers were identified and sent back to the field for verification. Initial observations were reviewed with fieldworkers and feedback given to optimize observations.

### Outcomes and analysis

#### Pharmacy summaries and available services

Summaries of data from the healthcare facilities surveys, including number of pharmacies and availability of medication equipment and staff, are presented as means, totals, or proportions without inferential statistics as they cover the whole population of facilities in the survey area. We informally summarise the products and services available at pharmacies in each area as described in Table 1. The list was not intended to be prescriptive or ideal and it includes items few, if any, pharmacies would reasonably have. However, it captures a broad view of the available services, equipment medicines, and staff available through local pharmacy outlets.

#### Pharmacy use and access

For analyses of data from the household and individual surveys, survey weights were calculated by bootstrapping the sampling procedure with 10,000 iterations, and averages (means, medians, or quantiles, etc.) are population weighted where they are reported.

We compared age-standardised visit rates to pharmacies with visits to other types of provider. This was accomplished by taking the overall outpatient healthcare visit rate, multiplying by the reported proportion of visits to the relevant provider with each age category, and then weighting using the INDEPTH LMIC standardized population [28]. To obtain 95% confidence intervals for these rates we bootstrapped this procedure 1000 times. We subdivided visits into those reported for “new conditions” and those for chronic or “existing conditions”. We also summarized the reasons for seeking care.

#### Observation of pharmacies

Qualitative observations were collated, read and reread by the team. Through comparison of data within and between sites we generated descriptions of different facility types.

## Results

### Pharmacies, products, and services available

**Fig. 1:**
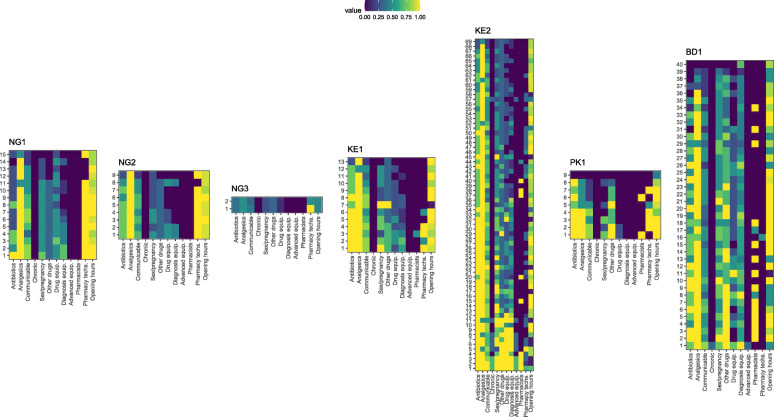
Graphical summary of the available products and services available in the pharmacies located within each slum area. Within each plot each pharmacy is represented by a separate row, and each column is a category as described in Table 1, with yellow indicating complete availability of all items in the category and dark blue none of the items available

**Fig. 2:**
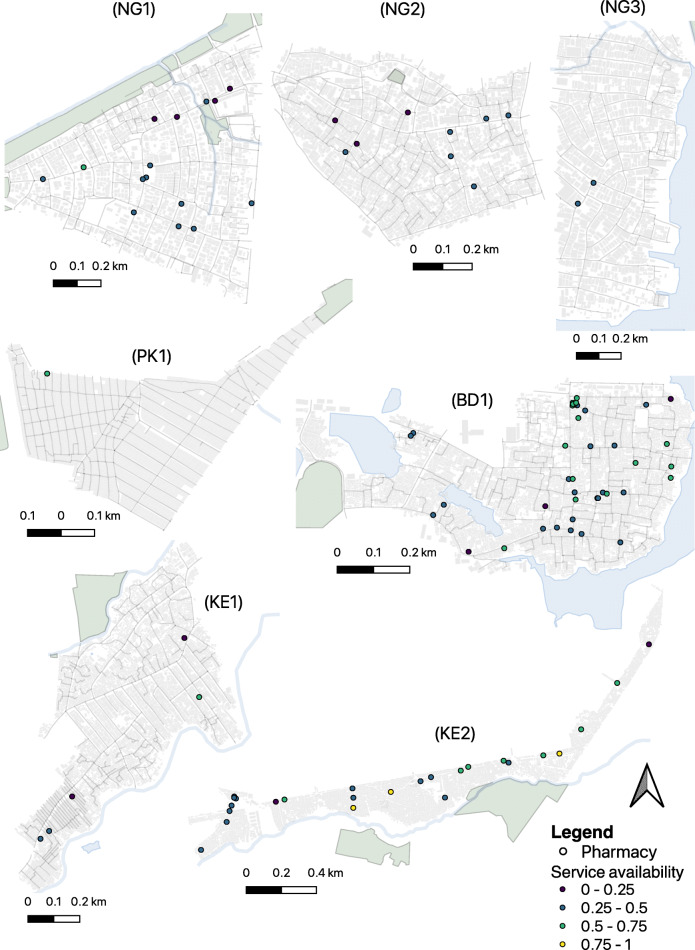
Locations of a selection of publicly identified pharmacies in each study site. Each location is coloured according to its overall summary score (see Table 1)

Overall, we identified 226 pharmacies across the seven slum sites (Table 2). Pharmacies were identified in all seven sites and included legally designated full-service pharmacies and several other classifications including patent medicine vendors (Nigeria), community and public health pharmacies (Kenya), medical stores (Pakistan), and medical shops (Bangladesh). Site NG1 had the most pharmacies per 1000 residents in the slum precinct and pharmacy technicians per capita, although all were lower designated “patent medicine vendors”; BD1 had the most qualified pharmacists per capita. Figure 1 summarises the available products, equipment, staff, and services available in the pharmacies in each slum site, and Fig. 2 shows a map of selected facilities that gave permission to show their location.

**Table 2:** Summary of pharmacies and pharmacy staff in each study site

	Nigeria			Kenya		Pakistan	Bangladesh
	NG1	NG2	NG3	KE1	KE2	PK1	BD1
Population	5800	5500	8100	24,000	45,000	33,500	60,000
Pharmacies total	22	15	6	14	68	9	92
Pharmacies per 1000	3.8	2.7	0.7	0.6	1.5	0.3	1.5
Pharmacists^a^ per 1000	0.5	0.0	0.0	0.0	0.3	0.1	0.6
Pharmacy technicians^a^ per 1000	2.9	2.7	0.6	0.2	0.7	0.2	0.0

^a^Either full-time or part-time

The proportion of pharmacies stocking each of the essential medicines is reported in Table S2 (Supplementary). Almost all essential medicines could be found within each slum site with the exception of those for chronic illness, including salbutamol (asthma), glibencamide (diabetes mellitus type 2), atenolol and captopril (hypertension), which were not available in the Nigerian sites and only partially available in the other sites. Oral contraceptives and condoms were available in all sites, but injectable contraceptives and pregnancy related medication were only available in KE2, PK2, and BD1, and only from a minority of pharmacies. Analgesia and antibiotics were widely available from most outlets in all countries.

Most pharmacies lacked basic equipment, including thermometers, weighing scales, and latex gloves (Table S3, Supplementary Information). Except for pharmacies in sites KE2 and BD1, the majority of pharmacies also lacked basic diagnostic equipment, such as blood pressure machine, weighing scales, and thermometer. Only site KE2 had pharmacies that scored 0.5 or better in every category (3 pharmacies of 68; see Table 1), and these were all attached to clinics.

### Qualitative pharmacy observations

Our definition of ‘pharmacy’ included a broad range of businesses and providers, from field-apprenticed drug vendors working in makeshift structures or stools to qualified pharmacists working in well-lit, ventilated and clean premises. Table 3 summarises our field observations.

**Table 3:** Qualitative observations of pharmacy characteristics

Characteristic	Summary
Premises	Premises ranged from small street stalls (e.g. 3ftx4ft) to larger independent medical stores (e.g. 10ftx12ft) and, in Kenya, Nigeria and Pakistan, dispensaries operating within clinics, health centres, and a sub-county hospital (KE1). Most were located along busy roads, within markets or near transport hubs and were often in close proximity to each other.
Goods and services	Depending on pharmacy size and scope, medical goods ranged from a few boxes of over-the-counter-drugs to shelves ‘loaded with tablets and syrups.’ Some also stocked herbal remedies. Most sold non-medical goods (e.g. soft drinks, soaps, sweets) and almost all offered mobile SIM and recharge services. In Kenya, Nigeria and Bangladesh, few displayed contact details, opening hours and authentication certificates. In Kenya and Bangladesh, some pharmacies were adjoined by a small doctor’s consulting chamber (e.g. 5ftx7ft). Drugs were often dispensed in small quantities (e.g. two tablets) in unlabeled containers, although some providers wrote or gave verbal directions to customers.
Privacy	Privacy was limited by space in all sites and customers often had to discuss and purchase items while standing on the street or a pavement step.
Cleanliness	In all sites, most facilities were located near to open drainage and ‘a lot of garbage’. Many sought to improve customer accessibility with wooden planks and elevated entrances. Inside, some stores were very dusty or ‘grimey’, others clean, well-lit and cooled by electric fans.
Opening hours	In all sites, most facilities stayed open late at night, 6–7 days per week, with owners on call after hours. For many, this was largely to accommodate ‘people who return late from work and need to get drugs especially pain relievers, most even call to book drugs and plead [for me to wait for them]’
Staff	In all sites, the owner/main provider was often referred to as ‘doctor’. Most drug vendors had undergone ‘in field’ training as apprentices and many in Nigeria and Bangladesh now employed apprentices themselves. A few mentioned medical backgrounds as nurses (NG, KE) or clinical medical officers (KE). Some were working elsewhere in ‘day jobs’ (in other health facilities or different industries outside of the site). During quiet times (e.g. mornings), young apprentices, family members or neighbours might be left to attend to occasional customers but at busier times and in larger facilities, teams of 3–6 assistants might work together to keep queues moving. In all sites, most providers were seen to be courteous and professional. Often a customer would first consult about their symptoms (no fee charged). Providers would then prescribe and sell treatment; or occasionally refer to a doctor/clinic.
Prices	Most transactions were in cash. In Nigeria, drug prices and quantities were negotiable, depending on ‘the severity of the case and the bargaining power’ of the customer. In Nigeria and Kenya, drugs were sold at government-subsidized rates in the primary healthcare centres. At the community primary healthcare centre in NG1, anti-malaria drugs were free and staff said they were always available (NG1). In Bangladesh, one pharmacy had a reimbursement arrangement to supply medicine at no cost to patients with prescriptions from a donor-funded clinic in the site.

### Pharmacy use

**Fig. 3:**
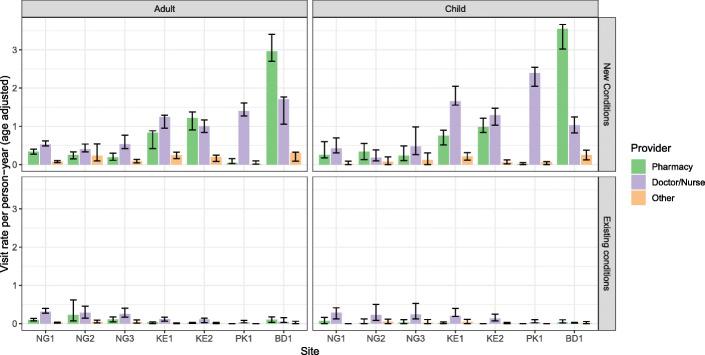
Pharmacy, doctor and nurse, and other consultations rates per person-year for adults and children (under 12) for both new and existing conditions with 95% confidence intervals

**Fig. 4:**
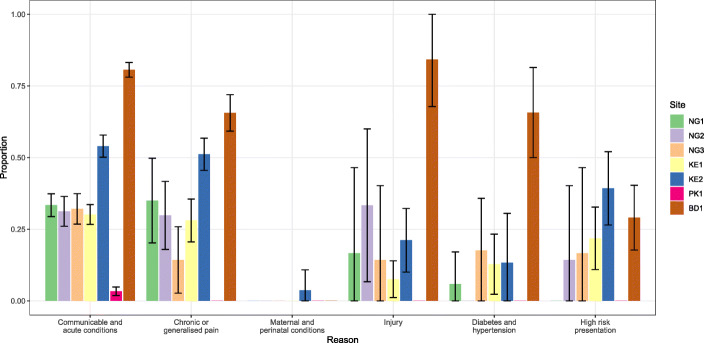
Proportion of reported visits by reason for seeking care that were to a pharmacy with 95% confidence intervals. “High risk presentation” includes chest pain, difficulty breathing, and stroke or sudden paralysis

Summary statistics of the surveyed individuals are reported in Supplementary Information. Figure 3 shows the visit rates to pharmacies alongside visit rates to other providers. Sites BD1 and KE2 were the only sites where visits to pharmacies were more frequent than visits to doctors or nurses. For example, in BD1 adults made on average three visits a year for new conditions compared to under 2 to doctors and nurses. Pharmacy visit rates in the other sites ranged from 0.1 (PK1) to 0.8 (KE1) visits per person-year. PK1 had a much lower visit rate to pharmacies than any other site as most respondents accessed medication and related services directly from a doctor in a clinic setting. Almost all visits were for new conditions. Visits for children were made at a comparable rate to adults. Figure 4 reports the proportions of reported outpatient visits to a pharmacy for different reasons for needing care. Acute conditions and pain were generally the categories most likely to result in a visit to a pharmacy. “High-risk” presentations, including chest pain, difficulty breathing, and stroke or sudden paralysis, accounted for between 1 and 5% of pharmacy visits. In NG1 and PK1 no visits for these conditions were reported to the pharmacy, however in the other sites anywhere from 10 to 40% of visits for these reasons were to a pharmacy.

### Cost and time to use pharmacy services

Table 4 reports the time and cost of a visit to a pharmacy in each site. The majority of all visits in all sites required less than 10 min of travel and less than 5 min of waiting. The highest spend per visit was highest in Nigeria where the median cost in the three sites ranged from Int$8 to 13. The lowest spend was in Kenya where the median cost was Int$ 3 in both sites.

**Table 4:** Median [IQR] travel and waiting times and spend per visit to pharmacies by study site

		Nigeria			Kenya		Pakistan	Bangladesh
		NG1	NG2	NG3	KE1	KE2	PK1	BD1
Spend per visit (Int$)		8 [3, 17]	8 [2, 13]	13 [5, 23]	3 [1, 10]	3 [1, 6]	5 [3, 9]	6 [2, 11]
Travel time (mins)		5 [5, 11]	5 [5, 10]	10 [5, 15]	10 [5, 20]	10 [5, 20]	5 [5, 10]	5 [4, 10]
Wait time (mins)		2 [0, 5]	2 [1, 5]	2 [1, 5]	1 [0, 5]	2 [0, 5]	4 [0, 5]	5 [3, 10]

## Discussion

We find that slum precincts are replete with drug sellers and pharmacists. As can be seen from Figs. 1 and 2, there is a high density of pharmacies in slums; over a half of people across all seven slums live within a ten-minute walk of a place selling medicines. That said, in four of our sites there was no qualified pharmacist working in the slum and in the remaining three sites the ratio of pharmacists to pharmacies varied from one in five to one in two. The availability of medicines from the WHO list of essential medicines was also very variable across vendors and in Nigeria and Kenya we found many of the essential medicines and basic equipment were not available, although we recognize our list of medication and equipment was not completely comprehensive. Many of the facilities were not legally permitted to sell certain medications, for example, patent medicine vendors in Nigeria were highly restricted in what drugs they could sell, while others were allowed to sell the drugs but did not have them in stock. These findings within slums are consistent with those found more generally in LMIC where poorly stocked outlets with poor service quality are widespread [10].

The high number of pharmacies in slums will come as no surprise to people familiar with informal settlements; they are a highly visible feature of the slum environment. But our study provides scientific confirmation that medicine outlets are plentiful. Medicines though are an out of pocket expense for people who live in slums [2]. Subsidized or third part payment is almost absent, except for in Kenya. And even when third party payments are available they tend to cover consultations not drug costs. The inference is that there must be sufficient private demand for medication in slums to support the high density of medicine sellers observed in this study.

We have described the typical costs spent on medicines above. In a companion study we show that medicines make up the largest proportion of outpatient costs exceeding those of consultations, even those that take place in the public sector. Given these costs, the lack of qualified staff, lack of privacy and cleanliness in many premises, and often poor availability of essential medication and equipment, we would concur that the plentiful pharmacy outlets are likely to provide a poor-quality service at high monetary cost [10, 11]. Our survey enquired after the last healthcare visit an individual made, so none of the pharmacy visits described here include a subsequent referral, even for potentially high-risk symptoms. Such a scenario leads to the hypothesis that medicine sellers are insufficiently knowledgeable. However, education is at best a partial solution according to a recent systematic review [15]. The review concluded that the interests of client and provider were poorly aligned leading to ‘market failure’. There is implicit recognition of this issue in countries rich and poor, including the four countries in our study, since medicine sales are regulated. The problem would appear to be one of enforcement which in turn is a function of money and political will. It is possible this is a reflection of the legal status of slums, however other evidence suggests the problems are not limited to these areas [10, 15]. Managing situations such as this is a perennial issue in development economics and it is to these methods that we believe we should turn with respect to improving the quality of pharmacy practice by theoretically sound and proportionate ways to gradually improve practice and services and ‘crowd out’ poor performance while rewarding good providers [29].

In developing a policy to improve pharmacy practice, it will be important to take into account one particular finding from our study; namely that there is a pressing demand for direct access to pharmacies in our sample – or at least relatively low cost, proximal, and rapid health care provision. This has two implications. First, pharmacies are a vast untapped resource for health prevention strategies and appropriate referral, with a potentially large public health role [7, 8]. This potential should be explored in the development and evaluation of policy. Second, providing public funding for access to standard health services is unlikely to extinguish demand to purchase some medicines directly since it has not done so in High Income Countries. The need to improve and capitalize on the medicine selling/pharmacy industry will thus remain an important policy objective notwithstanding extension of public funding and subsidies as well as improved regulation.

In conclusion, we show that the development of a viable market for medicines in urban areas extends into slums themselves but that there are grounds for thinking that this market does not operate effectively and needs carefully designed and enforceable regulation in addition to any education to fill knowledge gaps. Opinions regarding the role of community pharmacists has seen a shift in recent years from solely retailing products to taking on a substantial public health role comprising promotion, prevention, and disease management [8, 29]. The proximity of pharmacies to slums and often poor access to other types of care for slum residents face suggest that the shift to providing essential public health services should be explored in slum areas.

## Supplementary Information

**Additional file 1.**

## Data Availability

All data are available from the authors upon reasonable request.
