# Supplementary material for: Pharmacies in informal settlements: a retrospective, cross-sectional household and health facility survey in four countries
Source: BMC Health Serv Res. 2021 Sep 9;21:945. doi: 10.1186/s12913-021-06937-9 (PMC8431901; doi:10.1186/s12913-021-06937-9)
Supplement: Supplementary file 1 — Additional file 1. [file 12913_2021_6937_MOESM1_ESM.docx]

**SUPPLEMENTARY INFORMATION**

**TABLE S1** Summary statistics of the overall study sample

| **Variable** | | **Nigeria** | | | **Kenya** | | **Pakistan** | **Bangladesh** |
| --- | --- | --- | --- | --- | --- | --- | --- | --- |
|  |  | **NG1** | **NG2** | **NG3** | **KE1** | **KE2** | **PK1** | **BD1** |
|  |  | **All** | **All** | **All** | **All** | **All** | **All** | **All** |
| *Adults* | | | | | | | | |
| N | | 1,278 | 840 | 802 | 1,008 | 1,085 | 1,112 | 990 |
| Household size | | 4.2 (1.8) | 3.9 (1.7) | 4.6 (1.8) | 3.5 (2.1) | 2.5 (1.4) | 5.9 (2.8) | 4.3 (1.9) |
| Wealth quintile (%) | Bottom | 0 | 0 | 0 | 0 | 0 | 0 | 0 |
|  | Lower | 2 | 1 | 1 | 15 | 0 | 0 | 0 |
|  | Middle | 50 | 56 | 24 | 59 | 55 | 86 | 21 |
|  | Upper | 48 | 43 | 75 | 25 | 44 | 13 | 78 |
|  | Top | 0 | 0 | 0 | 0 | 1 | 1 | 1 |
| Monthly household expenditure (Int$) | Total | 326 (270) | 342 (274) | 560 (364) | 232 (136) | 243 (127) | 1,194 (683) | 463 (234) |
|  | Per person | 89 (75) | 95 (78) | 136 (101) | 84 (62) | 118 (81) | 225 (139) | 116 (58) |
| Age | | 41.5 (17.2) | 48.0 (17.0) | 42.0 (15.0) | 37.6 (13.6) | 34.3 (11.1) | 38.1 (13.7) | 34.6 (12.3) |
| Sex (% male) | | 45 | 49 | 47 | 46 | 46 | 50 | 53 |
| Education (%) | Primary/middle | 22 | 34 | 16 | 60 | 39 | 23 | 61 |
|  | Secondary | 46 | 55 | 54 | 34 | 51 | 62 | 31 |
|  | Tertiary | 24 | 9 | 32 | 6 | 8 | 14 | 8 |
| *Children (under 12)* | | | | | | | | |
| N | | 128 | 69 | 79 | 537 | 421 | 528 | 658 |
| Household size | | 4.9 (1.5) | 4.7 (1.4) | 4.9 (1.4) | 5.2 (1.8) | 4.4 (1.4) | 7.5 (3.0) | 4.9 (1.6) |
| Wealth quintile (%) | Lowest | 0 | 0 | 0 | 0 | 0 | 0 | 0 |
|  | Lower | 2 | 3 | 1 | 13 | 0 | 0 | 0 |
|  | Middle | 48 | 51 | 25 | 60 | 55 | 84 | 25 |
|  | Upper | 50 | 46 | 74 | 27 | 44 | 15 | 74 |
|  | Top | 0 | 0 | 0 | 0 | 0 | 1 | 1 |
| Monthly household expenditure (Int$) | Total | 365 (315) | 437 (310) | 579 (336) | 284 (29) | 276 (132) | 1,305 (112) | 472 (235) |
|  | Per person | 74 (58) | 93 (60) | 123 (68) | 56 (29) | 69 (37) | 184 (112) | 100 (46) |
| Age | | 6.9 (3.6) | 6.7 (3.7) | 6.5 (3.9) | 5.9 (3.7) | 5.6 (3.6) | 6.0 (3.7) | 5.5 (3.7) |
| Sex | | 44 | 57 | 43 | 55 | 48 | 53 | 52 |

**Table S2** Availability of essential medications in the formulation and dosage described in pharmacies (%)

| **Medications** | | **Nigeria** | | | **Kenya** | | | **Pakistan** | | | **Bangladesh** |
| --- | --- | --- | --- | --- | --- | --- | --- | --- | --- | --- | --- |
|  |  | NG1 | NG2 | NG3 | | KE1 | KE2 | | PK1 | BD1 | |
| Antibiotics | Ciprofloxacin (500 mg) caps or tabs | 36 | 73 | 33 | | 93 | 90 | | 78 | 80 | |
|  | Co-trimoxazole suspension for pediatric ID (8+40 mg/ml) | 14 | 67 | 33 | | 86 | 88 | | 89 | 49 | |
|  | Amoxicillin (500 mg) caps or tabs | 91 | 80 | 67 | | 100 | 99 | | 89 | 79 | |
|  | Ceftriaxone (1g/vial) injection | 18 | 0 | 0 | | 57 | 53 | | 78 | 28 | |
| Analgesics | Diclofenac (150 mg) caps or tabs | 86 | 100 | 67 | | 100 | 91 | | 44 | 87 | |
|  | Paracetamol suspension for pediatric patients (125 mg/ml) | 100 | 100 | 100 | | 93 | 99 | | 89 | 84 | |
| Non-antibiotic medication for communicable illnesses | Fluconazole (150 mg) caps or tabs | 18 | 33 | 33 | | 71 | 82 | | 44 | 75 | |
|  | Albendazole (400 mg) or medenazole (100 mg or 500 mg) chewable tabs | 73 | 20 | 33 | | 93 | 91 | | 56 | 72 | |
|  | Oral rehydration salts sachet | 100 | 100 | 67 | | 93 | 94 | | 89 | 97 | |
|  | Arteminisin combination therapy | 73 | 80 | 67 | | 57 | 74 | | 33 | 1 | |
|  | Isoniazid tablets (100-300 mg) | 0 | 0 | 0 | | 0 | 9 | | 11 | 3 | |
|  | Fansidar (SP, sulfadoxine, pyrimethamine) tab (500mg + 25mg) | 64 | 80 | 17 | | 71 | 38 | | 33 | 7 | |
| Medications for chronic illnesses | Salbutamol inhaler (0.1 mg/dose) | 9 | 0 | 17 | | 43 | 53 | | 44 | 55 | |
|  | Glibenclamide caps or tabs (5 mg) | 0 | 0 | 0 | | 57 | 43 | | 89 | 59 | |
|  | Atenolol (50 mg) caps or tabs | 0 | 0 | 33 | | 29 | 32 | | 78 | 45 | |
|  | Captopril (25 mg) or enalapril caps or tabs | 0 | 0 | 0 | | 43 | 22 | | 89 | 20 | |
|  | Simvastatin (20 mg) caps or tabs | 0 | 0 | 0 | | 7 | 6 | | 89 | 13 | |
|  | Efavirenz + lamivudine + tenofovir (TDF+3TC+EFV), or alternative combination ART | 0 | 0 | 0 | | 0 | 9 | | 0 | 2 | |
| Medications for sexual health, family planning, and pregnancy | Oxytocin (100-300 mg) injection | 9 | 0 | 0 | | 14 | 25 | | 22 | 11 | |
|  | Combined oral contraceptive pills | 68 | 33 | 33 | | 71 | 75 | | 44 | 92 | |
|  | Medroxyprogesterone (25 mg) injectable contraceptive | 0 | 0 | 0 | | 43 | 40 | | 11 | 33 | |
|  | Male condoms | 91 | 100 | 33 | | 86 | 90 | | 22 | 98 | |
| Other medications | Amitriptyline (25 mg) caps or tabs | 0 | 0 | 17 | | 36 | 19 | | 78 | 62 | |
|  | Omeprazole (20 mg) caps or tabs | 68 | 80 | 33 | | 100 | 99 | | 89 | 86 | |
|  | Vitamin A caps (200,000 IU) | 0 | 0 | 17 | | 7 | 24 | | 33 | 43 | |

**Table S3** Availability of medical equipment at pharmacies (%)

| **Equipment** | | **Nigeria** | | | **Kenya** | | | **Pakistan** | | | **Bangladesh** | |
| --- | --- | --- | --- | --- | --- | --- | --- | --- | --- | --- | --- | --- |
|  |  | NG1 | NG2 | NG3 | | KE1 | KE2 | | PK1 | BD1 | |  |
| Drug preparation and storage | Autoclave for sterilization | 0 | 0 | 0 | | 0 | 19 | | 0 | 10 | |  |
|  | Infusion kits for intravenous solution | 14 | 0 | 0 | | 14 | 22 | | 0 | 4 | |  |
|  | Refrigeration | 73 | 27 | 33 | | 86 | 87 | | 0 | 62 | |  |
|  | Latex gloves | 91 | 53 | 83 | | 7 | 24 | | 22 | 13 | |  |
| Basic diagnostic equipment | Microscope | 0 | 0 | 0 | | 14 | 21 | | 0 | 5 | |  |
|  | Slides | 0 | 0 | 33 | | 14 | 18 | | 0 | 12 | |  |
|  | Sphygmomanometer | 59 | 27 | 33 | | 21 | 54 | | 0 | 82 | |  |
|  | Stethoscope | 45 | 27 | 33 | | 29 | 60 | | 0 | 88 | |  |
|  | Adult weighing scale | 23 | 0 | 33 | | 14 | 26 | | 0 | 60 | |  |
|  | Weighing equipment for under-fives | 14 | 0 | 33 | | 0 | 25 | | 0 | 12 | |  |
|  | Thermometer | 64 | 27 | 33 | | 64 | 74 | | 0 | 98 | |  |
| Advanced equipment | X-ray machine | 0 | 0 | 0 | | 0 | 0 | | 0 | 0 | |  |
|  | Oxygen system/cylinders | 0 | 0 | 0 | | 0 | 10 | | 0 | 0 | |  |
|  | Cytoflowmeter | 0 | 0 | 0 | | 0 | 4 | | 0 | 3 | |  |
